# Special Issue: Parent–Child Interactions: Paths of Intergenerational Transmission of Psychopathological Risk

**DOI:** 10.3390/ijerph17249222

**Published:** 2020-12-10

**Authors:** Luca Cerniglia, Silvia Cimino

**Affiliations:** 1 Faculty of Psychology, International Telematic University Uninettuno, Corso Vittorio Emanuele II, 39, 00136 Rome, Italy; 2 Department of Dynamic and Clinical Psychology, Sapienza, University of Rome, 00186 Rome, Italy; silvia.cimino@uniroma1.it

The developmental psychopathology clinical and theoretical framework has proposed a bio-psycho-social model that integrates biological, environmental, social, and psychological factors to disentangle the underpinning mechanisms of the intergenerational transmission of psychopathological risk. It has been shown that parental psychopathological risk can predict maladaptive developmental outcomes in children, and evidence from animal studies has shown a direct link between the parental care environment and long-term effects on neural systems that regulate stress, emotional function, and neuroplasticity in offspring. These neurobiological and behavioral changes could be regulated by epigenetic mechanisms, and when parents are at risk of psychopathology, their caregiving capacity may decrease. This impairment in parental caregiving quality may act as a risk factor for negative outcomes in children. While several authors have posited that children have an innate predisposition toward bidirectional exchanges in different contexts (verbal interactions, feeding, and play), in those situations where caregivers and their offspring are incapable of mutually attuning and sharing sensitive interactions, the child may suffer poor emotional and behavioral self-regulation and face maladaptive symptoms over time. These negative outcomes may also be associated with (and predicted by) the temperamental characteristics of the child, such as irritability, negative moods, and irregular behavioral and biological patterns.

This Special Issue aimed to stimulate scientific discussion on the above topics, with particular attention to new methodological proposals for the assessment of interaction quality (e.g., new or improved observational methods, laboratory procedures, and diagnostic measures). Moreover, considering the current COVID-19 pandemic, the submission of papers addressing the possible effects of COVID-19 on parent–child interactions, children’s emotional/behavioral functioning, and possible outcomes in nurses and medical personnel was encouraged.

The Special Issue gathered several interesting research papers and reviews. Chirico, Andrei, Salvatori, Malaguti, and Trombini [1] recruited thirty parental couples and their children and longitudinally assessed parent–therapist alliances, parent–child interactions, and parenting stress subsequent to the Focal Play Therapy with Children and Parents intervention. The authors found that parent–child interactions significantly improved after the intervention, although parenting stress levels did not change between the two assessment points. This paper adds to scientific literature by informing about the role of parental engagement in preschool child-focused treatments. Moreover, this article informs clinical practice and contributes to the improvement of the quality of care for children and their families. In their paper, Hou, Chen, and Guo [2] used a transactional framework and a complex statistical cross-lagged model to explore the bidirectional links between depressive symptoms in caregivers and adolescents. They recruited 1644 adolescents and their parents and found that maternal and adolescent depressive symptoms were reciprocally interconnected, with the youth-driven effect being more robust than the mother-driven effect. Interestingly, youths’ depressive symptoms significantly predicted paternal depressive symptoms. These findings confirm a transactional effect of offspring characteristics and psychopathological risk on caregivers’ emotional and behavioral functioning. In a very interesting paper, Agostini et al. [3] described parents’ and infants’ interactive styles subsequent to assisted reproduction treatments and compared them with parent–infant interactions after spontaneous conception. They also evaluated the effect of infertility, treatment type, and previous assisted reproduction treatment attempts on interaction quality. The sample was composed of 25 couples who conceived with assisted reproduction treatments, 31 couples who conceived spontaneously, and their 3-month-old babies. The researchers evaluated free-play parent–infant interactions, and their results supported the importance of implementing agendas for more sensitive parenting styles. Pérez-de la Cruz and Ramírez [4] studied families with children with neuromotor disorders and evaluated parents’ perceptions and expectations about a physical therapy stimulation program. Their results showed the significance of accessing these types of support for children and families. In another interesting paper, Bao, Qu, Zhang, and Hogan [5] focused on children’s academic outcomes during the COVID-19 pandemic. Their statistical model predicted that reading ability in children during COVID-19 school closures would significantly decrease. Notably, the authors showed that children who had read a book during lockdown (even without formal education) would suffer a less marked reduction in their reading capacity. These results suggested that informal reading might constitute an effective strategy to maintain reading ability during school closures in future pandemic outbreaks. Coppola et al. [6] evaluated the correlation between caregivers’ narcissism and offspring narcissism. The sample was composed of 519 school-age children, and the results showed that parents’ narcissism was directly and positively related to overvaluation and the children’s narcissistic traits. Moreover, overvaluation moderately mediated the link between the fathers’ and children’s narcissistic traits. Moreover, these findings suggested that mothers and fathers communicate their narcissism to their offspring via differential pathways. Khoury, Rajamani, Bureau, Easterbrooks, and Lyons-Ruth [7] longitudinally studied the quality of parent–child interactions as risk indicators of the overall severity of maltreatment by age 18. One of the main results showed that hostile and withdrawn maternal interactions were associated with child maltreatment over time. This study could improve early identification and adapt preventive interventions for families with children at risk of maltreatment. In a sample of mother–child dyads, Speranza, Quintigliano, Lauriola, and Fortunato [8] tested a new clinician-report tool, the Parent–Child Relationship Scale, and evaluated parental and children’s individual contributions in parent–child interactions. They also assessed possible interactions between parents and children in terms of developmental psychopathology. Their results support the use of the new measure to assist with clinical diagnosis during early childhood. Trentini, Pagani, Lauriola, and Tambelli [9] recruited 20 mothers and 19 fathers during the third trimester of pregnancy and, through high-density electroencephalography, verified a possible link between neural responses to infant facial expressions and emotional self-awareness. Their results showed that parents presented similar activity in the areas involved in high-order socio-affective processes but different activity in premotor regions. Yu, Renzaho, Shi, Ling, and Chen [10] recruited a sample of 658 refugee children aged 5–17 years and their primary caregivers to evaluate the use of the Family Stress Model to verify the effect of family financial stress and parents’ levels of acculturation on children’s emotional and behavioral symptoms. Their results showed that this measure is effective and that financial stress and self-identity have effects on youths’ emotional and behavioral problems when mediated by caregivers’ psychological distress and hostile parenting. Sacchi, Facchini, Downing, and Simonelli [11] studied the possible application of a video-feedback intervention program, Primary Care Video Intervention Therapy, to support parenting. They found that this program alleviated maternal anxiety and supported parental capacity to recognize and talk about the infant’s developmental skills and regulatory abilities. Cimino, Marzilli, Tafà, and Cerniglia [12] recruited a community sample of 81 families with offspring aged between 19 and 28 months and explored possible links between the children’s DAT1 genotype, their psychological profiles, parental psychopathological risk, and the quality of mother–child and father–child feeding interactions. Their results showed that the 10/10-repeat polymorphism was associated with higher psychopathological risk. Another paper by the same research group (Cerniglia, Marzilli, and Cimino [13]) explored possible differences in children’s emotional–behavioral functioning, maternal psychopathologic risk, and the quality of mother–child feeding interactions in a sample of 100 dyads with offspring with different subgroups of Avoidant/Restrictive Food Intake Disorder. Their results showed differences between the groups in terms of children’s emotional–adaptive functioning, mothers’ psychological profile, and mother–child interactions during feeding. Finally, Wong et al. [14] presented a very informative review on demographic factors, needs and opportunities factors, family structures, and cultural–contextual structures associated with intergenerational resource transfer.
